# The Efficacy of Aloe vera Against Enterococcus faecalis as an Intracanal Medicament: A Systematic Review and Meta-Analysis

**DOI:** 10.7759/cureus.70140

**Published:** 2024-09-25

**Authors:** Seema H Bukhari, Dax Abraham, Shakila Mahesh

**Affiliations:** 1 Department of Conservative Dentistry and Endodontics, Manav Rachna Dental College, Faridabad, IND; 2 Department of Microbiology, Manav Rachna Dental College, Faridabad, IND

**Keywords:** aloe vera, calcium hydroxide, colony-forming units, enterococcus faecalis (e. faecalis), root canal treatment

## Abstract

Calcium hydroxide (CaOH) is commonly used as an intracanal medicament due to its antimicrobial properties; its antibacterial property depends on the release of hydroxyl ions. By analyzing experimental in vitro studies related to the research question, many studies carried out bacterial inoculation on extracted human teeth or laboratory Petri dishes. This review article seeks to assess the antimicrobial efficacy of Aloe vera (AV) against *Enterococcus faecalis* in comparison to CaOH as an intracanal medicament. After being retrieved from databases such as PubMed, Scopus, the Cochrane Library, and EBSCOhost, publications from 2013 to 2024 were screened against our inclusion criteria. As a result, seven papers were included in the systematic review and four in the meta-analysis. Using a meta-analysis (Stata software version 16.0, StataCorp LLC, College Station, TX) to compare colony-forming units (CFUs) formed by CaOH and AV, forest plots were created to record the specimen size, mean, and standard deviation value of the outcome in CFU, at 95% confidence intervals. AV exhibits bactericidal qualities that are equal to or comparable to those of CaOH. AV displayed a nonsignificantly reduced CFU count than CaOH in a meta-analysis (p > 0.05). In summary, AV exhibits antibacterial/antimicrobial capabilities against *E. faecalis* that are equal to or comparable to CaOH.

## Introduction and background

A vital stage in determining the prognosis and result of root canal therapy is cleaning and preparation of the root canal. The full eradication of bacteria resistant to antibiotics, like *E. faecalis*, remains a barrier for root canal therapy to be successful [1,2]. This gram-positive bacterium is most frequently detected in failed root canal treatments, occurring in 22%-77% of cases. *E. faecalis* can form biofilms in challenging conditions [3,4]. One of the most straightforward methods for removing and decreasing microbial flora from the root canal treatment is the administration of intracanal medications. The most widely used intracanal medication is calcium hydroxide (CaOH), which has a minimal effect on* E. faecalis *despite having a sufficient antibacterial range [5].

The adverse effects of synthetic pharmaceuticals have led to a recent increase in the trend toward the use of herbal remedies [6]. Patients with allergies to different chemical product components or contraindications are advised to use herbal treatments. Since bacteria that cause dental caries and periradicular pathology are becoming more resistant to antibiotics and synthetic drug side effects are becoming more common, researchers are looking into herbal alternatives for root canal treatment. Various plant extracts have been used for their antibacterial qualities [7].

According to estimates from the World Health Organization, 80% of people in underdeveloped nations use herbal medicine for basic medical needs. They are widely utilized in many underdeveloped countries, particularly those where they have long been a part of customary medical practices [8]. Aloe vera (AV)'s antibacterial, antiviral, antifungal, and anti-inflammatory qualities make it a popular herbal ingredient in mouthwash and toothpaste. The herb included phenolic chemicals called anthraquinone, which has an inhibitory impact on several oral infections such as *Candida albicans*, *Streptococcus pyogenes*, and* E. faecalis *[9,10]. Because AV is biocompatible and has not proven any toxicity to the periapex or adjacent tissues, it can be administered as an intracanal medication. AV gel's antibacterial qualities are assessed using a variety of techniques, including Agar diffusion, broth diffusion, and direct contact approach, against* E. faecalis* in both biofilm and planktonic states [11,12].

## Review

Protocol registration

Focused Question

Using the Population, Intervention, Comparison, and Outcome (PICOS) framework and the Cochrane Handbook for Systematic Reviews of Interventions as a guide, this review question was generated [13-15] using Population (P): single-rooted teeth that had been extracted and were infected with* E. faecalis,* Intervention (I): AV was utilized as an intracanal medication, and Comparison (C): CaOH, a regularly used intracanal medication, was employed as the comparison group. Colony-forming unit (CFU) counts were used to measure Outcome (O). Research (S) evaluates AV's antibacterial activity compared to CaOH.

Through mutual consent, the authors of the article designed the research topic by considering the availability, significance, and need for systemic reviews on relevant themes. As a result, this review compares the use of CaOH and AV as intracanal medications and includes in vitro studies. Thus, the last query was, "Is AV more effective than CaOH as an intracanal medication against *E. faecalis*?"

Literature Search Strategies

Scopus, PubMed, EBSCOhost, and Cochrane Library were included as major search engines for publication retrieval with the aid of the following keywords: "Aloe vera," "endodontics," and "*E. faecalis,"* and CaOH and the following combination of search terms was used to search articles: (Aloe OR "Aloe vera") AND (calcium hydroxide) AND (Endodontics OR "Regenerative Endodontics") AND *(faecalis*) using the All Fields function in PubMed, the Title-ABS-Key function in Scopus, and the Advance Search function with a Medical subheading in the Cochrane Library database. Articles were initially filtered according to the title and abstract of all papers that matched the inclusion criteria, and then, all articles meeting the inclusion criteria were considered relevant.

Inclusion Criteria

Studies performed on permanent single-rooted/premolars and human extracted teeth and in a laboratory (Petri dish) to compare the antibacterial activity of AV and CaOH as an intracanal medicament against *E. faecalis* were included, as shown in Table 1. After being retrieved from databases such as PubMed, Scopus, the Cochrane Library, and EBSCOhost, publications from 2013 to 2024 were screened.

**Table 1:** Office of Health Assessment and Translation tool for risk of bias and methodological quality assessment ++: definitely low risk of bias; +: probably low risk of bias; -: probably high risk of bias; NR: insufficient information; --: definitely high risk of bias

Vitro studies	Selection bias		Performance bias		Attrition/exclusion bias	Detection bias		Selective reporting bias	Other sources of bias
Study, country	1. Was random allocation present?	2. Was allocation to study groups adequately concealed?	5. Were the experimental conditions identical across study groups?	6. Were the research personnel blinded to the study group?	7. Were outcome data complete without attrition or exclusion from analysis?	8. Can we be confident in the intervention characterization?	9. Can we be confident in the outcome assessment?	10. Were all measured outcomes reported?	11. Statistical methods appropriate or did researchers adhere to the study protocol?
Patil et al. [11], India	NR	++	++	NR	++	++	_	++	+
Ghasemi et al. [16], Iran	NR	+	++	_	+	+	_ _	++	++
Eskandarinezhad et al. [17], Iran	_	+	+	NR	++	+	_ _	+	+
Varshini et al. [18], India	NR	++	+	NR	++	++	_	+	++
Bhardwaj et al. [15], India	NR	+	++	_	+	+	_ _	++	++
Ismail et al. [19], Malaysia	_	+	_	NR	++	++	_	++	++
Abbaszadegan et al. [3], Iran	NR	++	+	NR	++	++	_ _	+	+

Exclusion Criteria

In vivo studies, randomized controlled trials (RCTs) or non-RCTs, in vitro studies on animal or bovine teeth, studies including primary teeth (deciduous teeth) extracted from humans, case reports, case series, review articles, commentaries, unpublished papers, and letters to the editors were excluded. Thus, this review article compares an intervention (AV) with a control group, i.e., CaOH as an intracanal medicament, and includes in vitro studies. As such, the focused question was: "Is AV effective against* E. faecalis *as an intracanal medicament compared to CaOH?"

Quality Assessment of Included Studies

**Figure 1:**
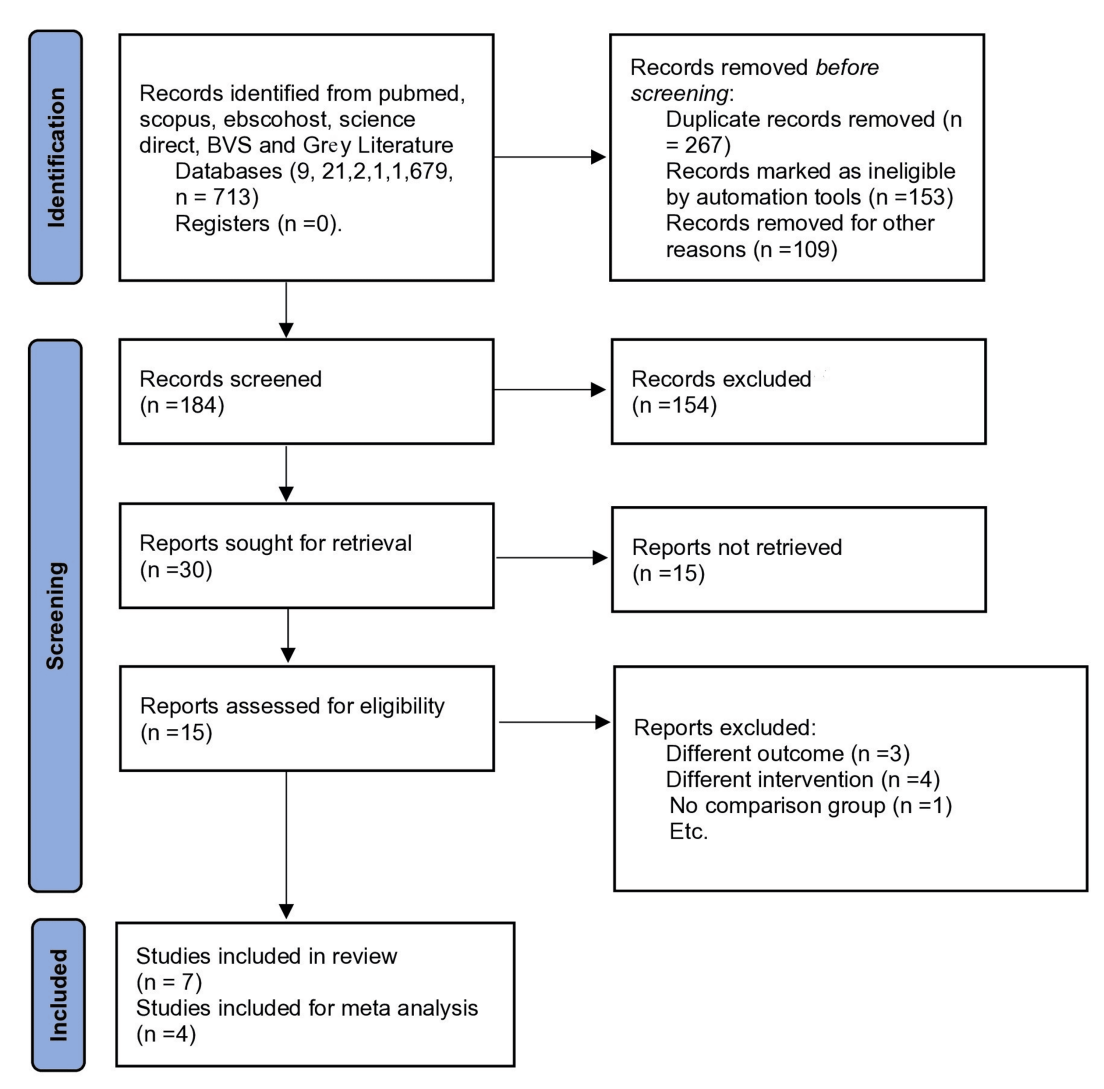
PRISMA 2020 flow diagram for systematic review and meta-analysis, which included searches of databases only PRISMA: Preferred Reporting Items for Systematic Reviews and Meta-Analyses

All the authors independently assessed the risk of bias for each included study. The systematic review was registered in the Open Science Framework and directed as per the Preferred Reporting Items for Systematic Reviews and Meta-Analyses (PRISMA) 2020 statement, as shown in Figure 1.

Data Collection/Extraction Process

Two authors SB and DA worked independently to extract the data, which included the type of study with the same or related factors. SB, DA, and SM agreed on the final data to be included, and disagreements were worked out through additional discussion. Only information pertinent to the inclusion criteria was retrieved wherever possible, and the study was disqualified if this kind of data disaggregation was not feasible. Two authors verified the accuracy of the extracted data.

Results

After the first database search, 713 studies were found, and 706 were removed after the titles and abstracts were examined. After removing duplicates, SB and DA removed reviews and studies that could not meet the eligibility requirements. The reviewers' discussions resolved the ambiguity in the research. The remaining seven studies [3,11,15-19] that focused only on the effectiveness of AV as an intracanal medication at various time intervals in removing* E. faecalis* from the root canal system were included in this systematic review.

Study Characteristics

Out of all the included studies, three studies were from Iran, one from Malaysia, and three were from India; six of the seven studies evaluated CFU using sound single-rooted teeth that had been inoculated with *E. faecalis*, while one study checked the zone of inhibition using the Mueller-Hinton agar. AV is the experimental group in this systematic review, and CaOH is the control group. Other intracanal medications, including Curcuma longa, mushrooms, castor oil, fresh lemon solution, *Morinda citrifolia* gel, 2% chlorhexidine, papain, Brazilian green propolis, Malaysian geopolis, and *Zataria multiflora*essential oil, were also investigated for their antibacterial effects. One of three methods was used to quantify the outcome, i.e., bacterial viability by confocal laser scanning microscopy analysis, antimicrobial sensitivity testing (AST), or bacterial culture by counting the (CFU) [16].

Characteristics of Individual Studies

The antibacterial activity of CaOH, extracts from mushrooms, AV, and *C. longa*, and intracanal medications against *E. faecalis* was compared and evaluated in a study by Patil et al. [11]. Twenty single-rooted specimens were biomechanically prepared, infected with *E. faecalis*, and cultured at 37°C for 21 days. Following incubation, a 10-fold dilution (up to a ratio of 1:105) was made, from which 0.1 mL was transferred and plated on the HiCrome^TM^ (Ludhiana, India) Mueller-Hinton agar medium. Before administering medication, CFUs were used to identify bacterial growth and assess the degree of contamination. Contaminated specimens were divided into five groups, with AV in group 3 and CaOH in group 1. All groups underwent 1, 7, and 14 days of incubation after placing the medications. The antibacterial efficacy was determined by comparing the percentage reduction in colony counts pre- and post-intracanal medication at three time intervals on the first, seventh, and fourteenth days. All of the medications demonstrated reduced* E. faecalis*, and the mean CFUs of each group decreased over time. Similarly, the CFUs were performed on the seventh and fourteenth day of incubation. The authors noted that when compared to *C. longa* and CaOH, AV showed a lower reduction in CFUs. The findings of a study by Bhardwaj et al., which claimed that AV and CaOH suppressed bacterial growth by 78.9% and 64.3%, respectively, are incompatible with these findings. The greatest CFU reduction was seen in *C. longa*, followed by CaOH, AV, and mushrooms [12].

Ismail et al. examined the efficacy of Malaysian propolis (MP) and AV and their combination against* E. faecalis* compared to intracanal medications containing CaOH. Three groups were created on Mueller-Hinton agar: MP, AV, and MP+AV. Egg-heart infusion agar was used for the inoculation of *E. faecalis*. For Mueller-Hinton agar plates, the bacterial density was adjusted to the McFarland standard 0.5 (1.5 × 10^8^ CFU/mL); 50 μL of MP, AV, MP+AV, CaOH, and 1% dimethyl sulfoxide (DMSO; control) were added to wells (6 mm). A total of 32 mg/mL of the active substance was present in each. After 18-24 hours of incubation at 37°C, the plate's inhibitory zones (mm) were measured. With a mean of 8.11 ± 0.015 mm, MP+AV had the biggest inhibition zone. MP (6.21 ± 0.046 mm), CaOH (5.51 ± 0.006 mm), AV (5.05 ± 0.012 mm), and 1% DMSO (0 mm) were the next in order. Regarding* E. faecalis,* CaOH exhibited a bigger inhibitory zone than AV [19].

Bhardwaj et al. conducted a study to evaluate the antibacterial effectiveness of CaOH and AV gel against *E. faecalis*. They separated the 180 single-rooted dentin blocks infected with* E. faecalis* into six groups. Antimicrobial evaluations were conducted after one, three, and five days of the root canals filling with CaOH (group 2) and AV gel (group 5). Samples of dentin were collected between 200 and 400 μm below the surface. The findings demonstrated that CaOH had substantial antibacterial activity on day 1, declined on day 3, and showed signs of recovery by day 5. On the first day, AV gel showed equal antibacterial efficacy; however, on the fifth day, efficacy steadily declined. Overall, CaOH exhibited 64.3% inhibition, and AV gel showed 78.9% inhibition throughout depths and time intervals, indicating the possibility of using AV gel as a substitute antimicrobial agent in RCTs.

Comparing CaOH, curcumin, and AV as intracanal medications against six-week-old *E. faecalis* biofilm was done by Eskandarinezhad et al. All medications considerably decreased *E. faecalis* after one week of use compared to the control group. Compared to controls, curcumin, CaOH, and AV showed 99.5%, 99.41%, and 98.79% of antibacterial activity, respectively. On the third day, AV's effectiveness was less than CaOH's, but by day 7, it had overtaken it. The results of this study point to AV's potential use in endodontic therapy as a useful supplement or substitute for traditional medications. It also highlights the plant's capacity to gradually increase antibacterial against persistent biofilms [20].

To eradicate* E. faecalis* from root canals, Abbaszadegan et al. compared the effectiveness of CaOH, AV, and *Z. multiflora* essential. They used 108 human single-rooted teeth that had undergone biomechanical and decoronation preparation. After being cultivated, *E. faecalis *was adjusted to 6.9 × 10^8^ (CFU/mL). Specimens were incubated for 21 days at 37°C and 95% relative humidity with intracanal medications. The roots were split into groups for 1, 7, and 14 days and given CaOH, *Z. multiflora* essential oil, or AV essential oil. After one and seven days of incubation, there was a statistically significant difference between the medications. After 14 days, there was no significant difference between them. For a 14-day extended contact period, the antibacterial efficacy of both medicinal herbs against *E. faecalis* was equal to that of CaOH.

Assessment of Risk of Bias

Using the Office of Health Assessment and Translation (OHAT) instrument, the systematic review assessed the methodological quality and risk of bias in seven publications. Group allocation and concealment (items 1 and 2) were among the 11 criteria of the tool that applied. Items 3 and 4 that did not pertain to in vitro and animal studies were eliminated. Good grades (items 5 and 6) were given to blinding and research characterization, indicating a sound approach. Items 7-9 presented all results as CFU or AST, while items 10 and 11 employed the proper statistical techniques. A tailored bias assessment criterion was used to classify most research as low risk. Meta-analysis was made possible despite the heterogeneity of the data by using the same CFU values and CaOH as a control in four different studies. This implies confidence in the ability to compare and synthesize findings from the included studies [21-23].

Meta-Analysis

**Figure 2:**
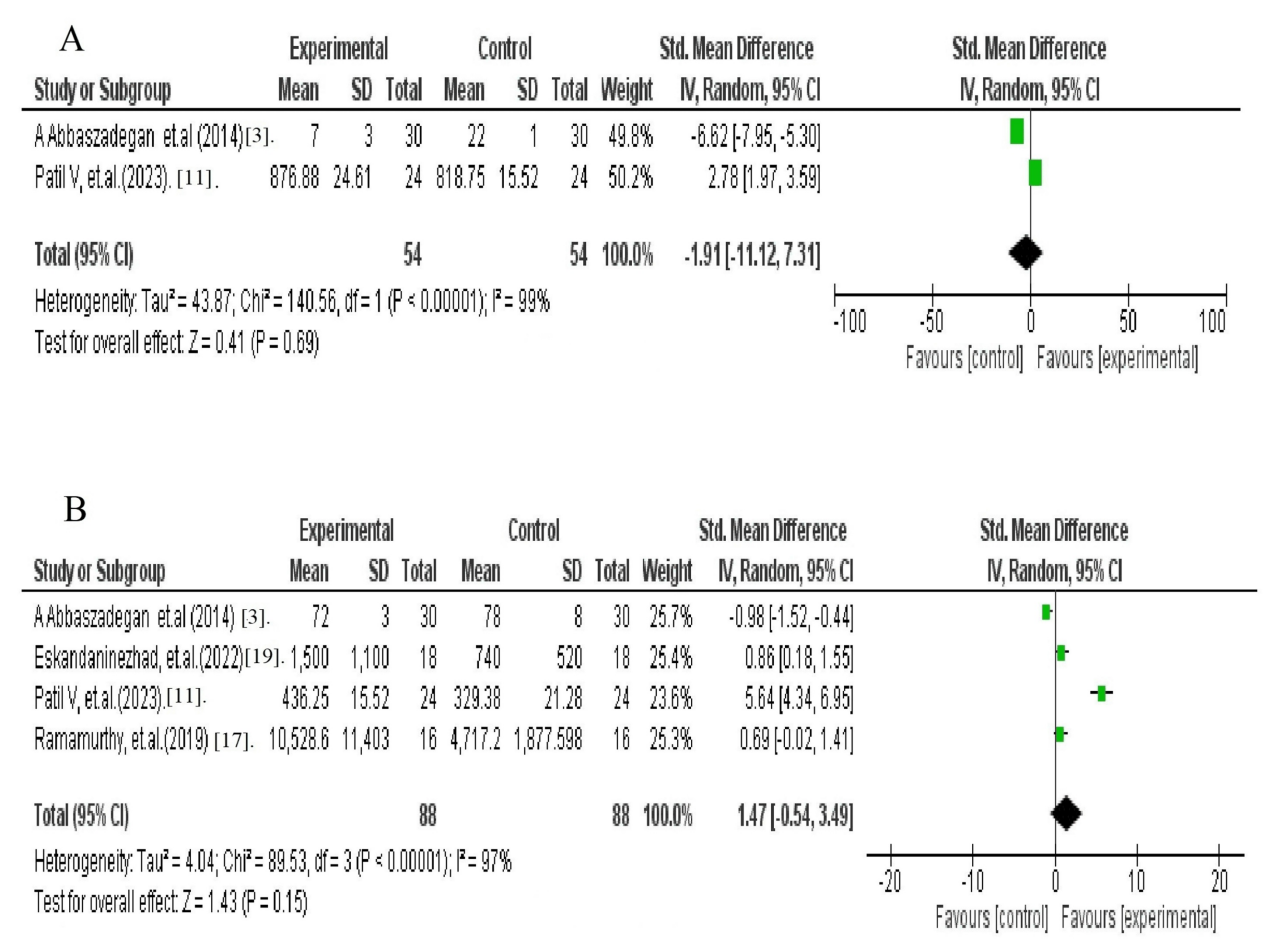
Forest plot showing mean and standard deviation between (A) AV and (B) CaOH at different time intervals (one day and one week) comparative in the reduction of E. faecalis CFU A and B illustrate the data on the first and seventh days, respectively AV: Aloe vera; CaOH: calcium hydroxide; CFU: colony-forming unit

The four studies included in the meta-analysis were selected based on their relevance, data availability, and methodological consistency for comparing CFUs of CaOH and AV. In a comparative analysis between AV and CaOH over intervals of one day and one week, AV demonstrated significant heterogeneity in the mean *E. faecalis* count in CFUs (H2 = 43.87, I2 = 99%), while CaOH exhibited H2 = 4.04, I2 = 97%. However, the overall mean differences in *E. faecalis* CFUs between AV and CaOH at these intervals were Overall = -1.91 (-11.12, 7.31); z =0.41; p = 0.69 and Overall = 1.47 (-0.54, 3.49); z = 1.43; p = 0.15), respectively. Analysis revealed that AV did not exhibit significant antibacterial action when compared to CaOH as an intracanal medication at various time intervals, as shown in Figures 2A, 2B. This difference was statistically nonsignificant [21-23].

Discussion

CaOH presents several limitations as an intracanal medicament, such as incomplete antimicrobial effectiveness and possible tissue irritation if extruded. Its gradual dissolution can reduce its efficacy, and necrotic tissue may affect its effectiveness. Additionally, the ideal duration of use remains uncertain, potentially impacting treatment outcomes. AV is becoming increasingly well known as an herbal remedy for various illnesses in dentistry. It has bactericidal qualities like CaOH and other intracanal medications used in dentistry [5]. Due to reports of its anti-inflammatory properties, AV is frequently used as a substitute medication in endodontics [22]. AV has been shown in numerous in vitro or ex vivo studies to be a potent intracanal medication; however, the length of exposure affects the medication's effectiveness.

There are seven studies in this systematic review, as mentioned in Figure 1. Six of these investigations evaluated CFUs at various time intervals, i.e., 1, 3, 5, 7, and 14 days on Petri dishes and mature biofilms at four and six weeks using permanent single-rooted premolars or human-extracted teeth, as in the study by Ghasemi et al. [16]. A laboratory Petri dish [19] was utilized in one investigation to assess the antibacterial activity of CaOH and AV as intracanal medications against* E. faecalis* at various time intervals and to determine the zone of inhibition using antimicrobial sensitivity.

AV's antibacterial efficacy as an intracanal medication against* E. faecalis* is not greater than or equal to that of conventional medications, according to the overall review and analysis of the included studies. However, it can be an alternative medication during endodontic treatments, particularly as an intracanal replacement for CaOH. Most included studies that measured bacterial counts in CFUs were included in the meta-analysis. Instead, they expressed their findings as a median percentage reduction. Table 2 lists the significant diverse results that resulted from the lack of reliable research with the same measurement units, medication exposure lengths, study designs, inoculation schedules, sample sizes, and sample media [17,20,21].

One-day and seven-day intervals are graphed on a forest plot to determine the significance of the hypothesis. When AV was compared to CaOH at different time intervals, there was nonsignificant heterogeneity in the overall mean of *E. faecalis* counts in CFUs, as the diamond shifted toward the control group on the first day later on the seventh day, diamond moved more in the direction of the experimental group. Thus, the results are not statistically significant, meaning that additional experimental research is needed to verify the veracity of the findings. The OHAT technique is used to evaluate research's methodological quality and bias risk [22-24]. The tool evaluates bias risk using 11 factors. As indicated in Table 2, studies that omitted items 1-3 from the OHAT tool were classified as low risk, those that reported items 4-6 as having a moderate bias, and those with more than six nonreported items as having a high bias. Low-risk bias is a classification applied to most of the included research [25-31].

Strengths and Limitations of the Study

This systematic review followed the PICOS criteria and PRISMA 2020 guidelines, ensuring a thorough and structured evaluation of AV versus CaOH as intracanal medicaments. It utilized a comprehensive literature search and assessed study quality using the OHAT tool. However, the review faced limitations due to significant heterogeneity among studies, which impacted the consistency of results. The meta-analysis revealed nonsignificant differences in antimicrobial efficacy between AV and CaOH, and the reliance on in vitro studies may not fully represent clinical effectiveness. Variations in study design and measurement methods further complicated the synthesis of findings. Future in vivo RCTs are necessary to confirm these results and evaluate the clinical applicability of AV.

AV has various dental applications due to its antimicrobial, anti-inflammatory, and wound-healing properties. It helps reduce gingivitis and treat oral mucosal lesions and is incorporated into mouthwashes and toothpaste to mitigate plaque formation. Its bioactive compounds enhance overall periodontal health and promote oral integrity.

Overall, the review and analysis of the included studies showed that AV's intracanal antibacterial efficacy against *E. faecalis* is neither higher nor equivalent to that of conventional medications. Nonetheless, it is unlikely that any intracanal medication has been able to eradicate bacterial populations. It is advised that more in vivo randomized controlled studies with comparable methodology and research designs be conducted to confirm AV's effectiveness as an intracanal medication against *E. faecalis.*

## Conclusions

AV had bactericidal activity against *E. faecalis* when administered as an intracanal medicament. The herb included phenolic chemicals called anthraquinone, which has an inhibitory impact on several oral infections such as *Candida albicans, Streptococcus pyogenes*, and* E. faecalis*. Because AV is biocompatible and has not proven any toxicity to the periapex or surrounding tissues, it can be administered as an intracanal medication. However, it is unlikely that any intracanal medication has been able to eradicate the bacterial count completely. Additional in vivo RCTs should be conducted using a parallel methodology and research design to confirm AV's effectiveness as an intracanal medication against* E. faecalis*.
